# Co-worker unprofessional behaviour and patient safety risks: an analysis of co-worker reports across eight Australian hospitals

**DOI:** 10.1093/intqhc/mzae030

**Published:** 2024-04-10

**Authors:** Ryan D McMullan, Kate Churruca, Peter Hibbert, Ling Li, Ruby Ash, Rachel Urwin, Antoinette Pavithra, Johanna I Westbrook

**Affiliations:** Faculty of Medicine, Health and Human Sciences, Australian Institute of Health Innovation, Macquarie University, Level 6, 75 Talavera Road, Sydney, NSW 2109, Australia; Faculty of Medicine, Health and Human Sciences, Australian Institute of Health Innovation, Macquarie University, Level 6, 75 Talavera Road, Sydney, NSW 2109, Australia; Faculty of Medicine, Health and Human Sciences, Australian Institute of Health Innovation, Macquarie University, Level 6, 75 Talavera Road, Sydney, NSW 2109, Australia; IIMPACT in Health, Allied Health and Human Performance, University of South Australia, GPO Box 2471, Adelaide, SA 5001, Australia; Faculty of Medicine, Health and Human Sciences, Australian Institute of Health Innovation, Macquarie University, Level 6, 75 Talavera Road, Sydney, NSW 2109, Australia; Faculty of Medicine, Health and Human Sciences, Australian Institute of Health Innovation, Macquarie University, Level 6, 75 Talavera Road, Sydney, NSW 2109, Australia; Faculty of Medicine, Health and Human Sciences, Australian Institute of Health Innovation, Macquarie University, Level 6, 75 Talavera Road, Sydney, NSW 2109, Australia; Faculty of Medicine, Health and Human Sciences, Australian Institute of Health Innovation, Macquarie University, Level 6, 75 Talavera Road, Sydney, NSW 2109, Australia; Faculty of Medicine, Health and Human Sciences, Australian Institute of Health Innovation, Macquarie University, Level 6, 75 Talavera Road, Sydney, NSW 2109, Australia

**Keywords:** unprofessional behaviour, co-worker reports, patient safety risks, organizational culture, hospitals, professional accountability

## Abstract

A key component of professional accountability programmes is online reporting tools that allow hospital staff to report co-worker unprofessional behaviour. Few studies have analysed data from these systems to further understand the nature or impact of unprofessional behaviour amongst staff. Ethos is a whole-of-hospital professional accountability programme that includes an online messaging system. Ethos has now been implemented across multiple Australian hospitals. This study examined reported unprofessional behaviour that staff indicated created a risk to patient safety. This study included 1310 Ethos submissions reporting co-worker unprofessional behaviour between 2017 and 2020 across eight Australian hospitals. Submissions that indicated the behaviour increased the risk to patient safety were identified. Descriptive summary statistics were presented for reporters and subjects of submissions about unprofessional behaviour. Logistic regression was applied to examine the association between each unprofessional behaviour (of the six most frequently reported in the Ethos submissions) and patient safety risk reported in the submissions. The descriptions in the reports were reviewed and the patient safety risks were coded using a framework aligned with the World Health Organization’s International Classification for Patient Safety. Of 1310 submissions about unprofessional behaviour, 395 (30.2%) indicated that there was a risk to patient safety. Nurses made the highest number of submissions that included a patient safety risk [3.47 submissions per 100 nursing staff, 95% confidence interval (CI): 3.09–3.9] compared to other professional groups. Medical professionals had the highest rate as subjects of submissions for unprofessional behaviour with a patient safety risk (5.19 submissions per 100 medical staff, 95% CI: 4.44–6.05). ‘Opinions being ignored’ (odds ratio: 1.68; 95% CI: 1.23–2.22; *P *< .001) and ‘someone withholding information which affects work performance’ were behaviours strongly associated with patient safety risk in the submissions (odds ratio: 2.50; 95% CI: 1.73–3.62; *P *< .001) compared to submissions without a patient safety risk. The two main types of risks to patient safety described were related to clinical process/procedure and clinical administration. Commonly reported events included staff not following policy or protocol; doctors refusing to review a patient; and interruptions and inadequate information during handover. Our findings indicate that unprofessional behaviour was associated with risks to patient safety. Co-worker reports about unprofessional behaviour have significant value as they can be used by organizations to better understand how unprofessional behaviour can disrupt work practices and lead to risks to patient safety.

## Introduction

Unprofessional behaviour among staff, ranging from incivility to physical assault, is detrimental to organizational culture and undermines both staff and patient safety [1–6]. A key component of professional accountability programmes designed to address unprofessional behaviour in hospitals is the use of an online reporting tool [7–11]. These systems allow hospital staff to submit reports describing disrespectful staff behaviour that undermines safety. Only a small number of studies have analysed co-worker reports to examine the types and perceived consequences of unprofessional behaviour in hospitals.

In a US study of a randomly selected subsample of 120 reports, a taxonomy was developed to summarize co-worker observations of physician unprofessional behaviour [12]. The taxonomy contained 22 codes organized into 4 domains of medical professionalism: competent medical care; clear and respectful communication; integrity; and responsibility. A majority of the 120 reports (60%) described disrespectful or offensive communication. A majority of reports was submitted by nurses (70%), and staff physicians were the most frequently reported (52%). However, these findings were from a small randomly selected subsample of reports from a total of 590 reports filed in a 16-month period at the Vanderbilt University Medical Center.

One large study of 13 653 patients and 202 surgeons found patients whose surgeons had a higher number of co-worker reports about unprofessional behaviour were at greater risk for medical and surgical complications compared to patients whose surgeons had fewer co-worker reports [13]. Studies looking at associations between the frequency of co-worker reports and particular patient outcomes are important in demonstrating the significant consequences of these behaviours. However, such studies do not shine light on the types of unprofessional behaviour which may be particularly problematic or contexts which may lead to increased patient safety risks. This hinders the development of targeted strategies. We aimed to undertake a detailed analysis of the content of co-worker reports of unprofessional behaviour where risks to patient safety were identified by the reporter. Our objectives were to (i) identify who made a submission where a patient safety risk associated with the reported unprofessional behaviour was identified, (ii) identify who were the subjects of the submissions with a patient safety risk, (iii) determine the types of unprofessional behaviour associated with a patient safety risk, and (iv) summarize the types of patient safety risks described in the submissions.

## Methods

### Design

We analysed hospital staff submissions from the Ethos online messaging system. The study was approved by the Human Research Ethics Committee of St Vincent’s Hospital Melbourne (reference: HREC/17/SVHM/237).

### Data source

#### Ethos messaging system

A whole-of-hospital professional accountability and culture change programme called Ethos was implemented across eight Australian hospitals between 2017 and 2020. The Ethos programme aims to encourage a culture of speaking up and address behaviours that undermine patient and staff safety. It also aims to promote professionalism by recognizing positive staff behaviours. Staff are trained to speak up about behaviours that may jeopardize patient safety [14, 15]. A key component of Ethos is a secure online messaging system accessible to all staff to make submissions about their experience of co-worker behaviours that either promote or undermine patient or staff safety. The messaging system was designed to provide an avenue for staff to report unprofessional behaviour in situations when they feel unable to speak up in the moment or to discuss with their supervisor. It was not designed to replace conversations between staff but to provide an additional mechanism that was not part of a formal complaint or disciplinary process. Messages are triaged, and trained peer messengers deliver informal feedback to individual staff members identified in messages to encourage reflection on behaviours that have been perceived negatively by others. Further details of the programme and feedback process have been reported elsewhere [10, 11]. Once logged into the Ethos messaging system, the user chooses the type of submission they wish to make: a feedback for recognition submission (reporting behaviour that positively impacted workplace culture) or a feedback for reflection submission (identifying behaviour that negatively impacted workplace culture). For all submissions, the user provides information including their professional group; the date and location of the incident; the name and professional group of the person who is the subject of the submission; a description of the event, including who was involved and any lead up to the observation; and any additional supporting evidence. Users who complete a feedback for reflection submission are also asked: ‘Do you think this event put PATIENT safety at risk?’ and ‘Do you think this event put STAFF safety at risk?’ If ‘Yes’, users are asked to briefly describe the risk.

Submissions from the Ethos online messaging system were downloaded, deidentified, and redacted to maintain anonymity by employees of the study hospitals. There was a total of 2504 Ethos submissions between 2017 and 2020 across the eight hospitals, of which 1310 were feedback for reflection submissions used in this analysis.

### Data coding

The 1310 feedback for reflection submissions had previously been coded for 26 unprofessional behaviours, described elsewhere [16]. This process involved four researchers independently coding 10% of the reflection submissions to assess consistency. Any disagreements were resolved through discussion, and once consensus was reached, one researcher coded the remaining reflection submissions.

Descriptions of the patient safety risks included in each of the reports were reviewed, and the types of patient safety risks were classified. We used a framework aligned with the WHO International Classification for Patient Safety (Supplementary Appendix A) for this process [17]. This framework consists of 14 incident types with each of these including subcategories. Coders (R.D.M. and K.C.) had several coding meetings in the initial stages with two experts (P.H. and R.A.) in using the International Classification for Patient Safety to confirm the approach to coding the submission descriptions. A random sample of 20% of submissions was double-coded, and Cohen’s κ statistics of the primary patient safety risk type was calculated to estimate inter-rater reliability. Discrepancies between the two coders were resolved by discussion with the two experts until consensus was reached. Cohen’s κ statistic of inter-rater reliability was substantial, κ = 0.73 [95% confidence interval (CI): 0.61–0.85]. One coder (R.D.M.) re-read and sub-categorized submissions that had been categorized into the three most frequent patient safety risk types.

### Data analysis

The analysis approach included descriptive statistical analysis and thematic analysis of submissions reporting unprofessional behaviour. Descriptive summary statistics were presented for (I) reporters of unprofessional behaviour and (II) subjects of submissions about unprofessional behaviour. Submissions per 100 staff for each professional group and related 95% CIs were calculated and stratified by the involvement of a patient safety risk (Yes/No). For the six most frequently reported unprofessional behaviours, logistic regression was applied to examine the association between each unprofessional behaviour and patient safety risk. Finally, the types of patient safety risks described and coded were summarized. Analyses were performed using Statistical Package for the Social Sciences (version 28).

Submissions categorized into the three most commonly occurring patient safety risk categories were thematically analysed to gain additional insights into these submissions. Excel (version 2302) was used to conduct the thematic analysis.

## Results

Of 1310 submissions about unprofessional behaviour, 395 (30.2%) indicated that there was a risk to patient safety.

### Staff who made a submission with and without a patient safety risk

Among all professional groups, nurses had the highest rate of submissions as a reporter of co-worker unprofessional behaviour with a patient safety risk (3.47 submissions per 100 nursing staff, 95% CI: 3.09–3.9) (Table 1). Non-clinical services staff had the lowest rate of these submissions (0.75 submissions per 100 non-clinical services staff, 95% CI: 0.49–1.14). Of the 913 submissions that did not pose a risk to patient safety, nurses had the highest rate of submissions (6.68 submissions per 100 nursing staff, 95% CI: 6.15–7.25), and medical staff had the lowest rate of submissions (2.42 submissions per 100 nursing staff, 95% CI: 1.93–3.05).

**Table 1.** Comparison of submissions made by each professional group with and without a patient safety risk.

	Submissions with patient safety risk		Submissions without patient safety risk	
Professional group of staff who made a submission, i.e. reporter	Submissions per 100 staff (95% CI)	*n*	Submissions per 100 staff (95% CI)	*n*
Nursing	3.47 (3.09–3.9)	273	6.68 (6.15–7.25)	526
Medical	1.6 (1.21–2.13)	47	2.42 (1.93–3.05)	71
Allied health & clinical services	1.62 (1.18–2.23)	37	5.77 (4.89–6.81)	132
Non-clinical services	0.75 (0.49–1.14)	22	2.69 (2.16–3.34)	79
Management & administrative	0.92 (0.56–1.5)	16	6.03 (5.01–7.26)	105
**Total**	**2.22 (2.02**–**2.45)**	**395**	**5.14 (4.82**–**5.47)**	**913^a^**

a Two submissions were excluded as they were not submitted by staff (e.g. volunteer).

### Subjects of submissions with and without a patient safety risk

Among all professional groups, medical professionals had the highest rate of submissions as a subject of submissions about unprofessional behaviour with a patient safety risk (5.19 submissions per 100 medical staff, 95% CI: 4.44–6.05) (Table 2), whereas non-clinical services staff had the lowest submissions (1.02 submissions per 100 non-clinical services staff, 95% CI: 0.72–1.46). In contrast, management and administrative staff had the highest rate of submissions as a subject of submissions about unprofessional behaviour without a patient safety risk (6.26 submissions per 100 management and administrative staff, 95% CI: 5.22–7.51). Non-clinical services staff had the lowest rate of submissions as a subject of submissions about unprofessional behaviour without a patient safety risk (3.51 submissions per 100 non-clinical services staff, 95% CI: 2.9–4.24).

**Table 2.** Professional groups of the subjects of submissions with and without a patient safety risk.

	Submissions with patient safety risk		Submissions without patient safety risk	
Professional group of staff who were the subject of the submission	Submissions per 100 staff (95% CI)	*n*	Submissions per 100 staff (95% CI)	*n*
Nursing	1.99 (1.71–2.33)	157	4.84 (4.39–5.34)	381
Medical	5.19 (4.44–6.05)	152	7.4 (6.51–8.41)	217
Allied health and clinical services	1.57 (1.14–2.18)	36	4.51 (3.73–5.44)	103
Non-clinical services	1.02 (0.72–1.46)	30	3.51 (2.9–4.24)	103
Management and administrative	1.15 (0.74–1.78)	20	6.26 (5.22–7.51)	109
**Total**	**2.22 (2.02**–**2.45)**	**395**	**5.14 (4.82**–**5.47)**	**913^a^**

a Two submissions were excluded as they were not submitted by staff (e.g. volunteer).

### Unprofessional behaviour associated with a patient safety risk

Table 3 reports the associations between unprofessional behaviour and patient safety on six of the most frequently reported unprofessional behaviours. Of all reflection submissions, ‘being spoken to rudely’ was the most frequently reported unprofessional behaviour. Logistic regression showed that a submission involving ‘opinions being ignored’ was 1.68 times more likely to be associated with a patient safety risk. A submission involving ‘someone withholding information which affects work performance’ was 2.50 times more likely to be associated with a patient safety risk. Submissions involving ‘having unjustified allegations made’ (odds ratio, 0.71; 95% CI: 0.47–1.08) or ‘being spoken to rudely’ (odds ratio, 0.73; 95% CI: 0.57–0.95) were less likely to be associated with a patient safety risk.

**Table 3.** The association between unprofessional behaviour and patient safety for the six most frequently reported unprofessional behaviours.

Unprofessional behaviour	Total number of submissions, *n* (%)	Submissions with a patient safety risk, *n* (%)	Submissions without a patient safety risk, *n* (%)	Odds ratio (95% CI; *P*-value)
Being spoken to rudely	705 (36.06)	202 (33.06)	503 (37.71)	0.73 (0.57–0.95; .017)
Being humiliated or ridiculed	346 (17.70)	85 (13.91)	261 (19.57)	0.73 (0.54–0.97; .031)
Opinions being ignored	332 (16.98)	125 (20.46)	197 (14.77)	1.68 (1.23–2.22; <.001)
Shouted at or being the target of anger	291 (14.88)	96 (15.71)	195 (14.62)	1.29 (0.96–1.72; .087)
Having unjustified allegations made	142 (7.26)	33 (5.40)	109 (8.17)	0.71 (0.47–1.08; .107)
Someone withholding information which affects work performance	139 (7.11)	70 (11.46)	69 (5.17)	2.50 (1.73–3.62; <.001)
**Total**	**1308^a^**	**395**	**913**	

Note: Submissions could include multiple unprofessional behaviours.

a Two submissions were excluded as they were not submitted by staff (e.g. volunteer).

### Patient safety risks described and thematic analysis

Across the 395 submissions with a patient safety risk, 9 (2%) included a blank patient safety risk description (i.e. no response), and 160 (41%) were excluded due to insufficient detail to be classified using the framework. This meant that over half of the submissions that included a patient safety description were classified according to the framework (*n* = 226/395, 57%). The two main types of patient safety risks described were related to clinical processes/procedures (*n* = 89/226, 39%) and clinical administration (*n* = 53, 23%) (Table 4). Of the 89 clinical process/procedure related risks, procedure/treatment/intervention (*n* = 29, 33%) and diagnosis/assessment (*n* = 29, 27%) were the most common subcategories. Of the 53 clinical administration-related risks, handover (*n* = 22, 42%) was the most common. See Table 5 for exemplar quotes for these subcategories. There were no descriptions of patient safety risks related to healthcare-associated infection, blood/blood products, nutrition, oxygen/gas/vapour, patient accidents, or infrastructure/buildings/fixtures.

**Table 4.** Principal patient safety risk categories and their frequency.

Patient safety risk categories	Number of reports, *n* (%)
Clinical process/procedure	89 (39.38)
Procedure/treatment/intervention	29 (32.58)
Diagnosis/assessment	24 (26.97)
General care/management	20 (22.46)
Test/investigations	12 (13.48)
Clinical orders	3 (3.37)
Screening/prevention/routine check-up	1 (1.12)
Detention/restraint	1 (1.12)
Clinical administration	53 (23.45)
Handover	22 (41.51)
Admission	13 (24.53)
Discharge	7 (13.21)
Transfer of care	7 (13.21)
Referral/consultation	2 (3.77)
Consent	2 (3.77)
Behaviour	28 (12.39)
Resources/organizational management	22 (9.73)
Medication/IV fluids	11 (4.87)
Documentation	8 (3.54)
Medical device/equipment	7 (3.10)
Falls	7 (3.10)
**Total**	**226**

Note: Subcategory percentages for clinical process/procedure and clinical administration were calculated based on the totals for each of these categories.

**Table 5.** Exemplar quotes for the common patient safety risk subcategories.

Patient safety risk category	Exemplar quotes
Clinical process/procedure Procedure/treatment/intervention	‘[deleted] was verbally aggressive throughout the surgical procedure towards me. [deleted] threw an instrument at me (however it missed and hit the floor) when I accidently handed the wrong instrument to what he wanted. Instrument hit patient before it fell to the floor. No injury to the patient, but a potential to do so.’ (submitted by a nurse)
Diagnosis/assessment	‘My intern made a referral to [deleted] for an elderly female due to concern for a C-spine injury. This was on my advice as the [deleted] registrar overnight. The intern waited until 7am to make the call to minimise disruption to Dr [deleted]. Dr [deleted] told the [deleted] intern his plan was retarded. He told the intern to remove a protective collar from the patient, allow her to move her neck and refer to another team (geriatrics). I called Dr [deleted] to ask that he see the patient this morning and assess the C-spine. I was not able to present the patient or reason for my call due to interruptions. Dr [deleted] told me our management was idiotic. He asked how long I have worked at [deleted], telling me he has been here since [deleted] and implying my inferiority. He made several attempts to discredit the referral by stating he would speak to the [deleted] consultant (I readily provided the consultants details - Dr [deleted] did not contact them).’ (submitted by a doctor)
Clinical administration Handover	‘[deleted] was as usually with her intimidating and condescending manner was asking questions while I was trying to handover but getting nowhere. As usual she wouldn’t let me speak and constantly talked over me and questioned me without letting me speak. A daily rigma-roll of this happening while handing over to [deleted]. The nurse in charge for night shift intervened and asked [deleted] to let me speak which she didn’t like and was rude back to the night in charge nurse as well who was only trying to help me knowing that I was being bullied and this happens all the time.’ (submitted by a nurse)

#### Procedure/treatment/intervention

Twenty-nine submissions described patient safety risks involving procedures/treatments/interventions. Commonly reported events included staff not following policy or protocol (*n* = 14); delays in a procedure (*n* = 5); interruptions (*n* = 4); staff ignoring others (*n* = 4); and disagreements among staff (*n* = 4); and outcomes included delays in the procedure (*n* = 5). One submission described a nurse being instructed by a surgeon to fulfil the responsibilities of both a surgical assistant and scrub nurse during the extraction of a ureteric stone. The surgeon verbally abused the nurse criticizing their inability to complete the tasks. A delay in a procedure was also commonly described whereby trained staff were not available to provide expertise for the treatment of patients. In one submission, a consultant was called to support an anaesthetic registrar to give sedation to a patient. The surgeon demanded to know why the patient had not been intubated. The surgeon stamped their foot and abruptly left the theatre. The surgery was delayed until appropriate airway staff became available.

#### Diagnosis/assessment

Twenty-four submissions described patient safety risks related to diagnosis and assessment. Frequently described circumstances included doctors refusing to review patients (*n* = 8); inadequate reviews (*n* = 6); doctors not responding to requests to review patients (*n* = 3); and delays in doctors reviewing patients (*n* = 2). Inadequate reviews of patients were commonly reported. One submission described a doctor being asked to review a patient by a nurse who was concerned that the patient was suicidal. The doctor did not introduce himself to the patient and asked three questions before abruptly leaving the room to answer a phone call whilst the patient was talking. The doctor refused to see the patient again and concluded the patient was not suicidal.

#### Handover

Staff described patient safety risks related to handover in 22 submissions. These related to adequate information not being provided during handover (*n* = 7); interruptions (*n* = 6); being ignored (*n* = 5); absent staff, staff walking away, or staff avoiding handover (*n* = 3); conflict over correct handover process (*n* = 3); and refusal to accept patients (*n* = 2). Interruptions commonly occurred whereby nurses were perceived to rudely ask questions to criticise or to complain that handovers were taking too long. One submission described a nurse’s handover being interrupted by two nurses making critical remarks because they perceived the bedside handover to be too extensive. Inadequate information during handover was also commonly described in the reports. One submission described a nurse providing a handover without details about patient symptoms, investigations completed, known results, or plans for ongoing care. When further information was requested, the nurse was dismissive and refused to provide additional details.

## Discussion

### Statement of principal findings

Our study explored the association between unprofessional behaviour and risks to patient safety described in submissions from a staff online messaging system. Nurses had the highest rate of submissions that identified a patient safety risk, whereas medical professionals had the highest rate as subjects of submissions for unprofessional behaviour with a patient safety risk. ‘Opinions being ignored’ and ‘someone withholding information which affects work performance’ were strongly associated with a patient safety risk. The three main types of patient safety risks described in the submissions were related to problems with procedures/treatments/interventions, diagnosis/assessment, and handovers.

### Strengths and limitations

One of the strengths of our study was the large sample emanating from eight hospitals, including private and public hospitals. Our findings extend previous studies by identifying patient safety risks associated with staff unprofessional behaviour. The Ethos online messaging system, like any reporting system, is limited by the quality of the submissions. Almost half of the submissions with a patient safety risk had to be excluded from thematic analysis due to insufficient detail.

### Interpretation within the context of the wider literature

Similar to previous studies, rates demonstrated that nurses were more likely to make submissions about unprofessional behaviour and medical staff were most often the subject of a submission compared to staff from other professional groups [7, 12–16]. Nurses and medical professionals are responsible for direct patient care and as such would be expected to make more submissions with a patient safety risk compared to staff from management and non-clinical services. It is possible that other factors influence staff reporting unprofessional behaviour via online messaging systems, including awareness and access to the system as well as perceptions about the effectiveness of the system [10].

‘Being spoken to rudely’ was the most frequently described unprofessional behaviour among submissions. ‘Opinions being ignored’ and ‘someone withholding information which affects work performance’ were strongly associated with a perceived risk to patient safety. Studies that have surveyed hospital staff and those that have examined co-worker reports about unprofessional behaviour have similarly found these behaviours to occur frequently [5, 12–19]. Reports made by staff in our study linked these types of co-worker communication to patient safety risks, and this finding is consistent with previous studies indicating that patient outcomes are worse when there is poor communication amongst staff as exemplified with behaviours such as ignoring opinions of clinical colleagues [20, 21].

Our study is the first to describe the types of patient safety risks described in co-worker reports about unprofessional behaviour. Previous studies have shown a link between surgeons with a higher number of co-worker reports and a higher risk of complications in their patients [13]. However, our study identified the types of patient safety risks related to staff unprofessional behaviour, with not following policy, poor communication, unavailability of staff, and interruptions all commonly described across events about procedure/treatment/intervention, diagnosis/assessment, and handover. These events illustrate the ways in which unprofessional behaviour may threaten patient care.

### Implications for policy, practice, and research

The patient safety risks described in the submissions are known to occur in hospitals [22, 23]. However, the relationship between these and unprofessional behaviour has rarely been examined. How unprofessional behaviour can be an antecedent to increased risks to patient safety were exemplified in the accounts provided by staff, particularly how such behaviour can undermine psychological safety [24]. Analyses of the reports also identified a number of situational factors and organizational characteristics beyond individual factors (e.g. personality) that may increase the propensity to engage in unprofessional behaviour, including high workload, time pressures, inadequate coordination, teamwork, supervision and leadership, and organizational constraints (e.g. lack of equipment or resources) [24, 25]. These potentially error-producing factors should be targets of organization-wide improvement strategies, along with focussing on efforts to change individual behaviour [26, 27]. A further illustration of the multifaceted drivers of unprofessional behaviour which interact in complex ways has been demonstrated in a recent realist review [24].

Unlike safety incident reporting systems, the Ethos messaging system was not designed to attempt to provide a comprehensive dataset of unprofessional behaviour but to deliver a tool for staff to provide informal feedback to co-workers. The Ethos programme aims to encourage open discussion, and the messaging system for unprofessional co-worker behaviours is designed as a last, not first, strategy to address unprofessional behaviour by colleagues. The dataset presents a unique window into the types of behaviours that staff find difficult to address in person, along with the perceived consequences of these behaviours. Our results highlight that strategies to improve safe co-worker communication should include a focus on behaviours such as withholding information and ignoring opinions and their contributions to increased safety risks to patients. This evidence is much needed to inform more effective intervention programmes [28].

Martinez *et al*. [12] developed a taxonomy of unprofessional behaviour to facilitate the process of systematically and reliably tracking the types and patterns of unprofessional behaviour amongst hospital staff. We have shown that by using the framework aligned with the WHO International Classification for Patient Safety [17], risks to patient safety can be described and analysed to further understand their nature and their relationship with unprofessional behaviour. The safety literature emphasizes that multiple information sources are important for incidents to be adequately characterized and understood, as each information source is likely to detect different types of incidents [29, 30]. Healthcare organizations may consider using co-worker reports about unprofessional behaviour as an additional information source to characterize risks to patient safety.

## Conclusion

Our findings highlight some of the specific mechanisms by which unprofessional behaviour was associated with perceived increased risks to patient safety. Unprofessional behaviours were reported to occur in contexts where staff did not follow policy or protocol, they refused to review patients, and they interrupted or provided inadequate information during handover. Unprofessional behaviour can jeopardize psychological safety, which is essential for clinical staff to raise concerns and engage in open discussion when assessing patients or completing handovers. Situational and organizational factors such as inadequate coordination and lack of resources are understudied and require further investigation to determine how they drive unprofessional behaviour and impact patient safety. Online reporting tools can provide novel datasets that can be used by organizations to strengthen strategies to promote professionalism and improve patient safety.

## Supplementary Material

mzae030_Supp

## Data Availability

Data cannot be shared for ethical/privacy reasons. The data underlying this article cannot be shared publicly due to the human research ethics committee conditions to maintain the privacy of staff who completed reports.
