# Supplementary material for: Co-worker unprofessional behaviour and patient safety risks: an analysis of co-worker reports across eight Australian hospitals
Source: Int J Qual Health Care. 2024 Apr 10;36(2):mzae030. doi: 10.1093/intqhc/mzae030 (PMC11025673; doi:10.1093/intqhc/mzae030)
Supplement: mzae030_Supp [file mzae030_supp.zip › suppl_data/Appendix A.docx]

**Appendix A**

Framework aligned with the WHO International Classification for Patient Safety

| **Incident Type** | **Process** |
| --- | --- |
| Clinical Administration | Handover |
|  | Appointment |
|  | Waiting list |
|  | Referral/Consultation |
|  | Admission |
|  | Discharge |
|  | Transfer of care |
|  | Patient identification |
|  | Consent |
| Clinical Process and Procedure | Screening/prevention/ routine check-up |
|  | Diagnosis/assessment |
|  | Procedure/ treatment/ intervention |
|  | General care/management |
|  | Tests/investigations |
|  | Specimens/results |
|  | Detention/restraint |
|  | Clinical orders |
|  | Deterioration |
| Documentation | Orders/requests |
|  | Charts/medical records/ assessments/ consultations |
|  | Check lists |
|  | Forms/certificates |
|  | Instructions/information/policies/procedures/guidelines |
|  | Labels/Stickers/Identification Bands/Cards |
|  | Reports/results/images |
| Healthcare Associated Infection | Bloodstream |
|  | Surgical site |
|  | Abscess |
|  | Respiratory |
|  | Intravascular cannulae |
|  | Infected prosthesis/site |
|  | Urinary drain/tube |
|  | UTI |
|  | Cellulitis |
|  | Conjunctivitis |
|  | VRE |
|  | MRSA |
|  | Gastroenteritis |
|  | Wound |
| Medication/IV Fluids | Prescribing |
|  | Preparation/dispensing |
|  | Presentation/packaging |
|  | Delivery |
|  | Administration |
|  | Supply/ordering |
|  | Storage |
|  | Monitoring |
| Blood/Blood Products | Pre-Transfusion Testing |
|  | Prescribing |
|  | Preparation/Dispensing |
|  | Delivery |
|  | Administration |
|  | Storage |
|  | Monitoring |
|  | Presentation/Packaging |
|  | Supply/Ordering |
| Nutrition | Prescribing/Requesting |
|  | Preparation/Manufacturing/ Cooking |
|  | Supply/Ordering |
|  | Presentation |
|  | Dispensing/Allocation |
|  | Delivery |
|  | Administration |
|  | Storage |
| Oxygen/Gas/ Vapour | Cylinder Labelling/Color Coding/PIN Indexing |
|  | Prescription |
|  | Administration |
|  | Delivery |
|  | Supply/Ordering |
|  | Storage |
| Medical device/ equipment | Medical device/equipment |
| Behaviour | Staff/Pt Behaviour |
| Patient accident | Pt accident |
| Falls | Fall involving cot |
|  | Fall involving bed |
|  | Fall involving chair |
|  | Fall involving stretcher |
|  | Fall involving toilet |
|  | Fall involving therapeutic equipment |
|  | Fall involving stairs/steps |
|  | Fall involving being Carried/Supported by Another Individual |
| Infrastructure/Buildings/Fixtures | Infrastructure/building fixture |
|  | signage |
| Resources/Organisational management | Resources/organisational management |
